# The complete mitochondrial genome of *Nysius fuscovittatus* (Hemiptera: Lygaeidae)

**DOI:** 10.1080/23802359.2020.1827062

**Published:** 2020-10-05

**Authors:** Ya Cao, Hui-Ting Wu, Min Li, Wen-Ting Chen, Ming-Long Yuan

**Affiliations:** a State Key Laboratory of Grassland Agro-Ecosystems, Lanzhou University, Lanzhou, People's Republic of China; b Key Laboratory of Grassland Livestock Industry Innovation, Ministry of Agriculture and Rural Affairs, Lanzhou, People's Republic of China; c National Demonstration Center for Experimental Grassland Science Education, Lanzhou University, Lanzhou, People's Republic of China; d College of Pastoral Agricultural Science and Technology, Lanzhou University, Lanzhou, People's Republic of China

**Keywords:** Insects, Lygaeidae, mitochondrial DNA, phylogenetic analysis

## Abstract

Here, we sequenced and annotated the complete mitochondrial genome (mitogenome) of *Nysius fuscovittatus* (Hemiptera: Lygaeidae). This mitogenome was 14575 bp long, including 13 protein-coding genes (PCGs), 22 transfer RNA genes (tRNAs), 2 ribosomal RNA unit genes (*rrnL and rrnS*), and a putative control region. All genes were arranged in the same order as that of most true bugs. Eleven PCGs started with a typical ATN, and the remaining two PCGs started with TTA (*nad4L*) and TTG (*cox1*). The *N. fuscovittatus* mitogenome with an A + T content of 76.42% showed a positive AT-skew (0.15) and a negative GC-skew (–0.15). With the exception of *trnS1* that lacked the dihydrouridine arm, all tRNAs had a typical cloverleaf secondary structure. Phylogenetic analysis based on the concatenated nucleotide sequences of the 13 PCGs showed that *N. fuscovittatus* clustered with other three Lygaeidae species.

Lygaeoidea (Hemiptera: Heteroptera) includes over 4600 described species in approximate 15 families. Most Lygaeoidea species are phytophagous, whereas a few are predators. Currently, the phylogenetic relationships among families within Lygaeoidea are still controversial (Yuan et al. 2015; Zhang et al. 2019). Here, we sequenced the complete mitochondrial genome (mitogenome) of *Nysius fuscovittatus* from the family Lygaeidae by high-throughput sequencing.

Adult specimens of *N. fuscovittatus* were collected from Menyuan County, Qinghai Province, China, in October 2019 (101°21′04″ E, 37°62′48″ N). Samples (voucher number MY-59) have been deposited in the State Key Laboratory of Grassland Agro-Ecosystems, College of Pastoral Agricultural Science and Technology, Lanzhou University, Lanzhou, China. The total genomic DNA was extracted from a single specimen using a DNeasy Tissue Kit (Qiagen).

The complete mitogenome of *N. fuscovittatus* was a circular molecule of 14,575 bp in size and has been deposited in NCBI (GenBank accession number MT242601). This mitogenome contained 13 protein-coding genes (PCG), 22 transfer RNA genes (tRNA), 2 ribosomal RNA unit genes (*rrnL* and *rrnS*), and a putative control region. The mitochondrial gene arrangement of *N. fuscovittatus* was identical to that of most true bugs (Yuan et al. 2015; Zhang et al. 2019). Gene overlaps were observed at five gene junctions and involved a total of 16 bp. The longest overlap (7 bp) existed between *atp6* and *atp8* and the overlapped seven nucleotides were ATGATAA. In addition to the putative control region, a total of 109 nucleotides were dispersed 10 intergenic spacers, ranging in size from 1 bp to 67 bp. The longest intergenic spacers existed between *trnH* and *nad4*.

The nucleotide composition of the *N. fuscovittatus* mitogenome was significantly biased toward A and T. The A + T content was 76.42% (A = 43.78%, T = 32.64%, C = 13.54%, G = 10.05%). This mitogenome presented a positive AT-skew (0.15) and a negative GC-skew (–0.15), as found in most insect mitogenomes. The *rrnL* was 1269 bp long with an A + T content of 78.09%, and the *rrnS* was 714 bp with an A + T content of 76.33%. Among the 13 PCGs, *atp8* (84.62%) was higher in A + T content, whereas *cox3* (71.45%) and *cox1* (71.19%) were the lowest. Eleven PCGs started with a typical ATN codon, whereas the remaining two PCGs started with TTA (*nad4L*) and TTG (*cox1*). Three PCGs terminated with a complete end codon TAA (*atp8*, *nad4* and *nad4L*), whereas the remaining ten terminated with an incomplete stop codon TA– or T—. All of the 22 tRNAs, ranging from 61 bp (*trnA*) to 73 bp (*trnK*), had a typical cloverleaf secondary structure, except for *trnS1* which lacked the dihydrouridine arm.

Phylogenetic analysis was performed with the concatenated nucleotide sequences of 13 PCGs from 12 Lygaeoidea species and 1 Pyrrhocoroidea species (outgroup). The Bayesian inference was performed with MrBayes 3.2.6 (Ronquist and Huelsenbeck 2003) on the CIPRES Science Gateway 3.3 (Miller et al. 2010). The phylogenetic tree indicated that *N. fuscovittatus* clustered with other three Lygaeidae species and Lygaeidae was sister to Berytidae and Geocoridae (Figure 1).

**Figure 1.**
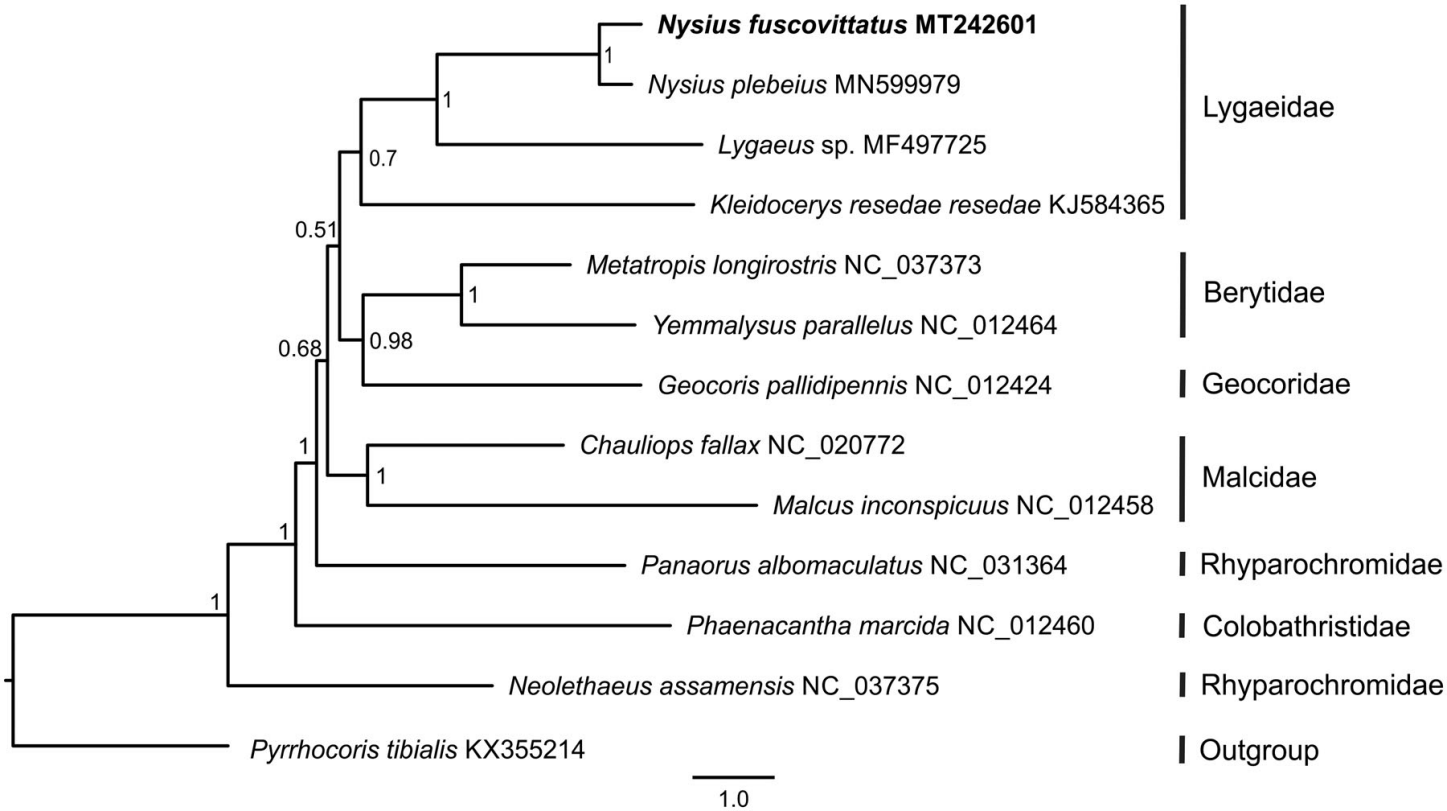
Bayesian phylogenetic tree inferred from the concatenated nucleotide sequences of 13 mitochondrial protein-coding genes of 12 Lygaeoidea. Numbers at the nodes are posterior probabilities.

## Data Availability

The data that support the findings of this study are available in GenBank of NCBI at https://www.ncbi.nlm.nih.gov, reference number MT242601.

## References

[CIT0001] Miller MA, Pfeiffer W, Schwartz T. 2010. Creating the CIPRES Science Gateway for inference of large phylogenetic trees. Proceedings of the Gateway Computing Environments Workshop (GCE); 2010, Los Angeles. p. 1–8.

[CIT0002] Ronquist F, Huelsenbeck JP. 2003. MrBayes 3: bayesian phylogenetic inference under mixed models. Bioinformatics. 19(12):1572–1574. 12912839 10.1093/bioinformatics/btg180

[CIT0003] Yuan ML, Zhang QL, Guo ZL, Wang J, Shen YY. 2015. Comparative mitogenomic analysis of the superfamily pentatomoidea (Insecta: Hemiptera: Heteroptera) and phylogenetic implications. BMC Genomics. 16(1):460. 26076960 10.1186/s12864-015-1679-x PMC4469028

[CIT0004] Zhang QL, Feng RQ, Li M, Cao Y, Guo ZL, Zhang LJ, Luo FZ, Yuan ML. 2019. The complete mitogenome of *Pyrrhocoris tibialis* (Hemiptera: Pyrrhocoridae) and phylogenetic implications. Genes. 10(10):820. 10.3390/genes10100820 PMC6826757 31635273

